# Antioxidant properties of bee propolis and an important component, galangin, described by X-ray crystal structure, DFT-D and hydrodynamic voltammetry

**DOI:** 10.1371/journal.pone.0267624

**Published:** 2022-05-18

**Authors:** Francesco Caruso, Molly Berinato, Melissa Hernandez, Stuart Belli, Christopher Smart, Miriam Rossi

**Affiliations:** Department of Chemistry, Vassar College, Poughkeepsie, New York, United States of America; Beijing Foreign Studies University, CHINA

## Abstract

Propolis is produced by honeybees and used to seal their hives for defensive purposes and has been used in ethnopharmacology since ancient times. It is a lipophilic material containing a large collection of naturally produced plant organic molecules, including flavonoids. The flavonoid galangin is consistently found in propolis, independent of the hive geographical location and its X-ray crystal and molecular structure is reported. The antioxidant scavenging of superoxide by galangin and propolis is here presented. Using a cyclic voltammetry technique developed in our lab, we show that galangin is an excellent scavenger of the superoxide radical, perhaps even better than quercetin. Our results show that galangin displays a Superoxide Dismutase (SOD) function. This is described experimentally and theoretically (DFT). Two modes of scavenging superoxide are seen for galangin: (1) superoxide radical extraction of H atom from the hydroxyl moieties located in position 3 and 5 of galangin, which are also associated with proton incorporation defining the SOD action; (2) π-π interaction among several superoxide radicals and the galangin polyphenol ring that evolve towards release of O_2_ and H_2_O_2_. We describe these two actions separately as their relative sequence, and/or combination, cannot be defined; all these processes are thermodynamically spontaneous, or subjected to mild barriers.

## Introduction

Bee propolis is a viscous natural product produced by honeybees to seal their hive. Although its chemical composition varies according to environmental conditions, geographical location and available plant populations, the biological health benefits of propolis long have been appreciated throughout the world as part of traditional medicines. For instance, ancient Egyptians used propolis in embalming procedures because of its antimicrobial properties [1, 2]. The extensive health properties arise from the compounds in propolis. Notwithstanding the variations described earlier, bee propolis consistently contains a high concentration of phenolic compounds, including flavones. In a recent study, galangin and chrysin were determined to be the most abundant polyphenols of a brown propolis extract [3]. Galangin, 3,5,7-trihydroxyflavone, is recognized to have significant beneficial biological activities such as anticancer [4, 5], antioxidant [6, 7]. Galangin is also found in plants such as *Alpinia officinarum* Hance (Zingiberaceae), or lesser galangal, and has been used as a spice and in herbal remedies for a variety of ailments in Ayurvedic and Chinese medicine for centuries [8, 9].

In this work, we have studied the antioxidant activity of galangin and of samples of bee propolis from Wappingers Falls in the Mid-Hudson region of New York State, USA, using the Rotating Ring Disk Electrode (RRDE) cyclic voltammetry technique that was developed in our laboratory. This method permits us to evaluate the superoxide radical scavenging activity of the pure compound, galangin, as well as samples of bee propolis. Additionally, we obtained high quality crystals of galangin, suitable for X-ray diffraction studies and we describe the molecular geometry and intermolecular interactions. Earlier structure-activity studies have suggested that the 2,3-double bond in conjugation with the 4-oxo group in the flavonoid structure is a major determinant of flavonoid antioxidant activity in mitochondria [10]. Many studies relating to flavonoid and polyphenolic antioxidant potential have been reported [11–18]. In our study, we focus on the slight structural differences between galangin and chrysin, as they can be related to the experimental determination of superoxide scavenging. Moreover, using computational results from dispersion-corrected Density Functional Theory (DFT-D), we study interactions among galangin and superoxide radicals. We suggest a mechanism for scavenging of the superoxide radical anion by galangin, and bee propolis, in agreement with our cyclovoltammetry findings.

### Experimental section

#### X-ray

Galangin (Indofine Chemical Co, Hillsborough, NJ, USA) was recrystallized from ethanol by slow evaporation. Table 1 shows the crystal data. Data collection was taken with a Bruker Smart APEXII single crystal X-ray diffractometer, with a Charge Coupled Device Detector and the experiment was done at 125 K using a cold liquid nitrogen stream from Oxford cryosystems. The X-ray source emitted Mo_Kα_ radiation at 0.71073 Å. We used the SHELX program to determine and refine antioxidant crystal structure [19], and input the X-ray data into the MERCURY program to produce images of the molecules and crystal packing [20]. Crystal data of galangin have been deposited at the Cambridge Structural Database (CSD) and are available at https://www.ccdc.cam.ac.uk/structures/? using Identifier CCDC number 2123390.

**Table 1:** Crystal data of galangin●H_2_O.

Empirical formula	C_15_ H_10_ O_5_ ∙H_2_O	
Formula weight	280.25	
Temperature	125(2) K	
Wavelength	0.71073 Å	
Crystal system	Triclinic	
Space group	P-1	
	a = 3.8308(13) Å	α = 80.139(4°.
	b = 11.080(4) Å	β = 86.049(4°.
	c = 14.741(5) Å	γ = 83.351(4°.
Volume	611.5(4) Å3	
Z	2	
Density (calculated)	1.522 Mg/m3	
Absorption coefficient	0.117 mm-1	
F(000)	292	
Crystal size	0.23 x 0.19 x 0.05 mm3	
Theta range for data collection	1.40 to 27.48°.	
Index ranges	-4<=h<=4, -14<=k<=14, -19<=l<=19	
Reflections collected	7372	
Independent reflections	2769 [R(int) = 0.0259]	
Completeness to theta = 27.48°	99.6%	
Absorption correction	Empirical	
Max. and min. transmission	0.9942 and 0.9735	
Refinement method	Full-matrix least-squares on F2	
Data / restraints / parameters	2769 / 0 / 238	
Goodness-of-fit on F2	0.972	
Final R indices [I>2sigma(I)]	R1 = 0.0364, wR2 = 0.0993	
R indices (all data)	R1 = 0.0454, wR2 = 0.1066	
Largest diff. peak and hole	0.335 and -0.213 e.Å-3	

#### RRDE measurement of antioxidant activity

Materials used to determine the antioxidant activity of galangin and propolis were tetrabutylammonium bromide (TBAB; Sigma Aldrich) and 99% anhydrous Dimethyl Sulfoxide (DMSO; Sigma Aldrich. Galangin (Indofine Chemical Co., Hillsborough, NJ, USA). Propolis was provided by a co-author (C. Smart), from his own hive in the Hudson Valley. The 0.1 M TBAB/DMSO solution was used to produce electric current and enhance the occurrence of redox reactions. Antioxidant activity was measured via the hydrodynamic voltammetry technique with a rotating ring disk electrode (RRDE). The equipment used in this experiment was an MSR electrode rotator with CE and ETL marks, together with a WaveDriver 20 benchtop USB from Pine Instrumentation. The main electrode tip was an E6RI ChangeDisk with a rigid gold ring and gold disk (Au/Au) insert. Before and after each experiment, 0.3μL Alumina suspension was used to clean the disk electrode tip (Allied High Tech Products, Inc, Rancho Dominguez, CA, USA) on a moistened polishing microcloth to eliminate potential film formation. A Platinum (Pt) reference electrode and Pt counter electrode were also used in this experiment. All electrodes were obtained from Pine Research Durham, NC, USA. [21]. Cyclic voltammagrams were run using a Solartron SI 1287 Potentiostat/galvanostat (Solartron Analytical, Oakridge, TN, USA) controlled through Coreware© software.

The antioxidant activity of galangin was determined based on its superoxide radical scavenging ability that was measured using the protocol developed in our lab [22]. A stock solution of galangin, 0.02 M, in anhydrous DMSO was used in trials, whereas 0.335 g of propolis were dissolved in 10 ml of DMSO and used as stock solution. For the experiment, the electrolytic cell was bubbled for 5 minutes with a dry O_2_/N_2_ (35%/65%) gas mixture to establish its dissolved oxygen level. The Au disk electrode was then rotated at 1000 rpm while the disk was swept from 0.2 V to -1.2 Volts and the ring was held constant at 0.0 Volts, the disk voltage sweep rate was set to 25 mV/s. In summary, 4 runs plus blank were performed in the RRDE experiment for galangin to determine the antioxidant activity with equal addition of 5 μL of galangin stock solution, whereas 5 aliquots, were added for propolis study.

Results from each run were collected on Aftermath software and represented as voltammograms showing current vs. potential graphs that were later analyzed using Microsoft Excel. In an RRDE voltammetry experiment, the generation of the superoxide radicals occurs at the disk electrode while the oxidation of the residual superoxide radicals (that have not been scavenged by the scavenger) occurs at the ring electrode.

Reaction 1: Reduction of molecular oxygen at disk electrode

$${\text{Disk}\;\text{current}\;\text{O}}_{2}+\text{e-}⇌{\text{O}}_{2}{•}^{-}$$ (1)

Reverse Reaction 2: Oxidation of superoxide radicals at the ring

$${\text{Ring}\;\text{current}\;\text{O}}_{2}{•}^{-}⇌{\text{O}}_{2}+\text{e-}$$ (2)

Thus, the rate at which increasing concentrations of galangin scavenged the generated superoxide radicals during the electrolytic reaction was determined by obtaining the quotient of the ring current and the disk current (percent value) at each concentration. These values were denoted as the *collection efficiency* of the scavenger at different concentrations. Using Microsoft Excel, collective efficiency values were plotted against the corresponding concentrations of galangin to produce a graph illustrating the effect of increasing concentrations of galangin on the scavenging of superoxide radicals in the electrolytic solution. Ultimately, the slope of the curves served as a quantitative measure of the antioxidant activity of galangin and propolis.

#### Computational experiments. Antioxidant activity of galangin studied using DFT

Calculations were performed using programs from Biovia (SanDiego, CA, USA). Density functional theory (DFT) code DMol3 was applied to calculate energy, geometry, TS and frequencies implemented in Materials Studio 7.0 [23]. We employed the double numerical polarized (DNP) basis set that included all the occupied atomic orbitals plus a second set of valence atomic orbitals, and polarized d-valence orbitals [24]; the correlation generalized gradient approximation (GGA) was applied including Becke exchange [25], plus BLYP-D correlation including Grimme’s correction when van der Waals interactions were involved [26]. We used the DNP basis set, which consists of a double-numeric quality basis set for approximately 2 atomic orbitals for each one occupied in free atom (DN), plus basis with polarization functions, that is, functions with angular momentum one higher than that of highest occupied orbital in free atom.

Calculations were performed with DMSO solvent inclusion, using the continuous model of Dmol^3^ [27]. We used the DNP basis set, which consists in a double-numeric quality basis set for approximately 2 atomic orbitals for each one occupied in free atom (DN) plus basis with polarization functions, that is, functions with angular momentum one higher than that of highest occupied orbital in free atom. All electrons were treated explicitly and the real space cutoff of 5 Å was imposed for numerical integration of the Hamiltonian matrix elements. The self-consistent field convergence criterion was set to the root mean square change in the electronic density to be less than 10^−6^ electron/Å^3^.

No human, animal or cell studies were used in research described in this manuscript.

## Results and discussion

### X-ray study

**Fig 1:**
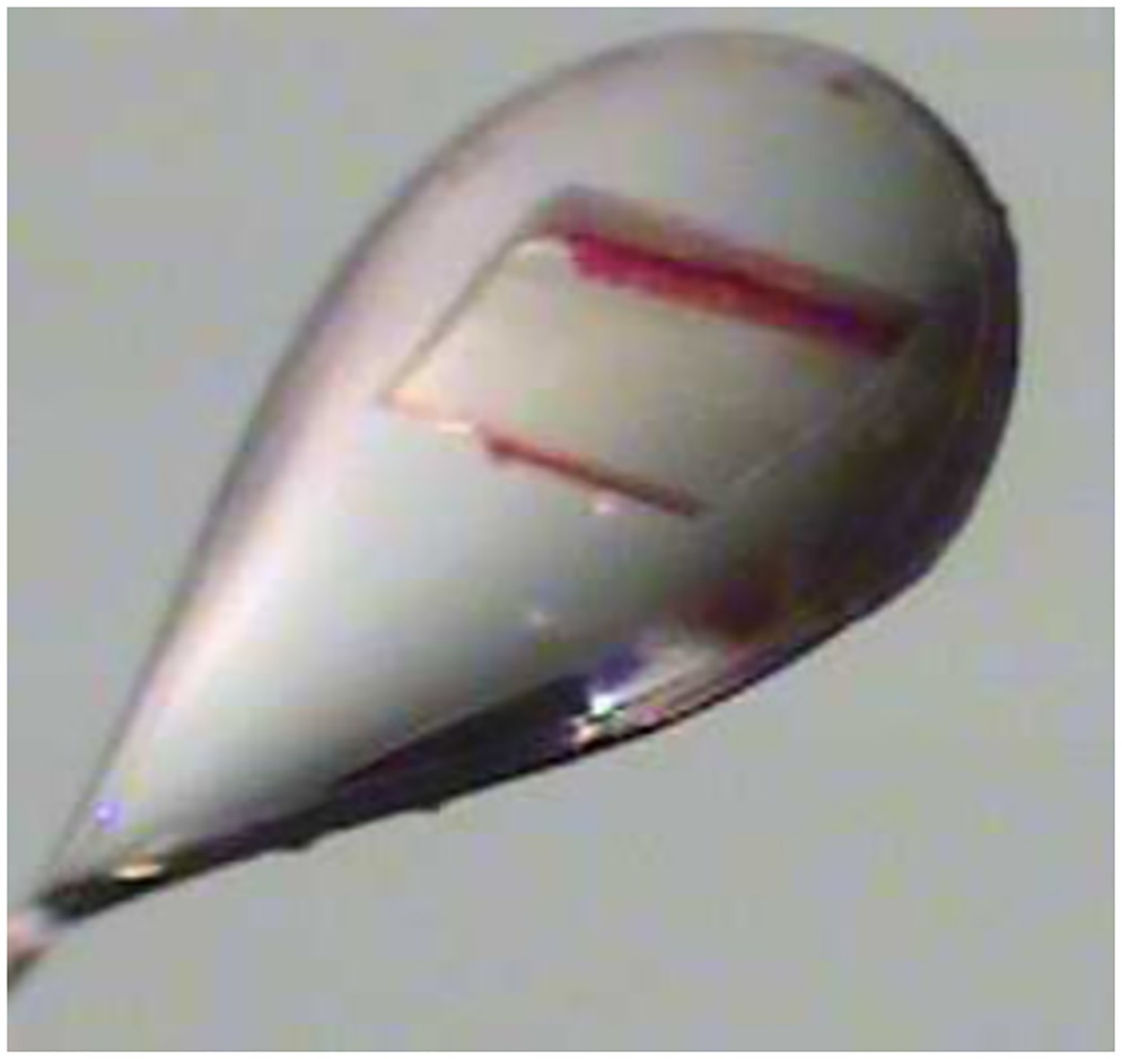
Galangin data crystal showing the color variation.

**Fig 2:**
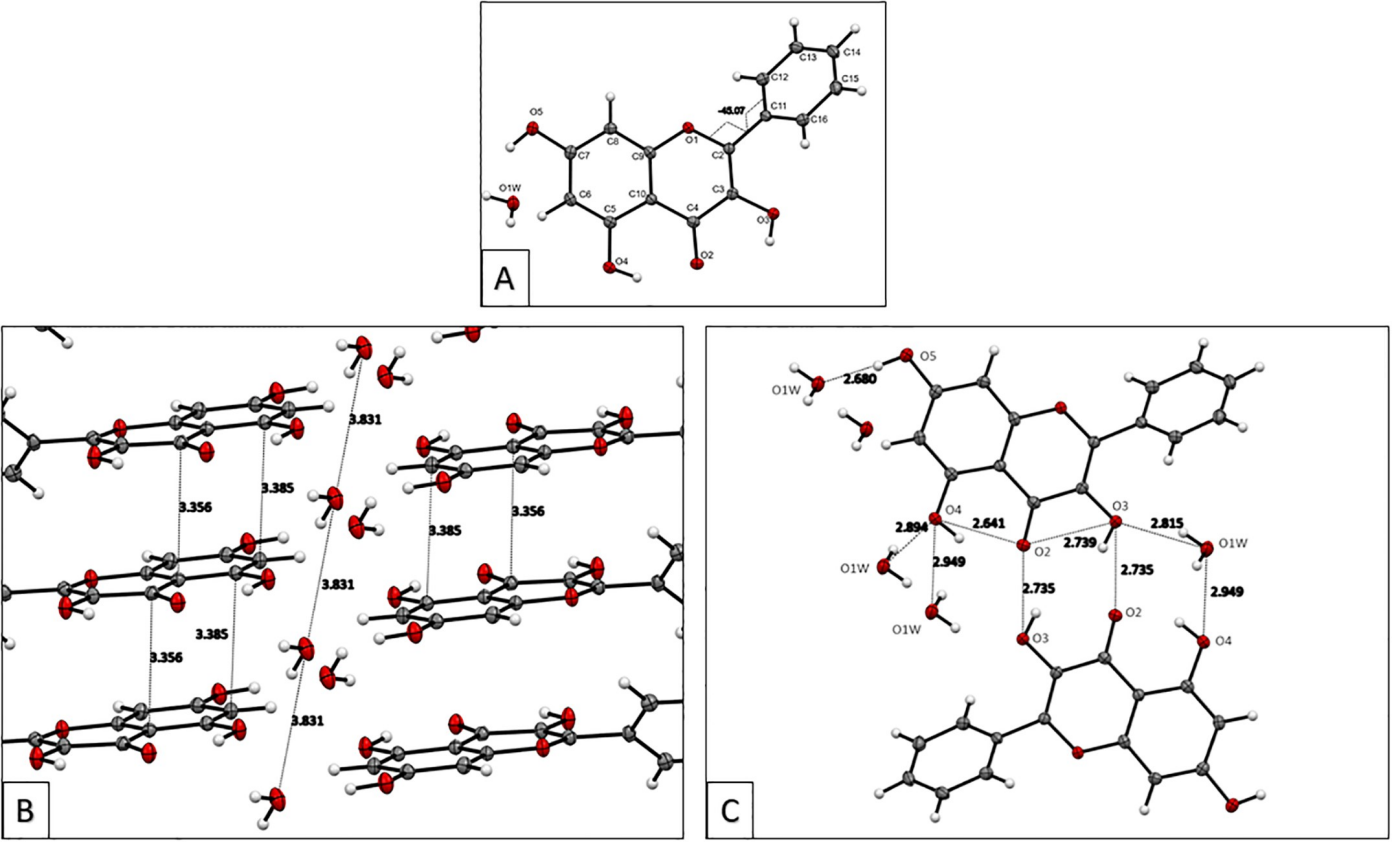
A: The asymmetric unit of galangin monohydrate with atom labeling and the torsion angle about the C2-C11 bond at -45.1(2°; B: Offset stacking interactions among galangin molecules (about 3.35–3.38 Å). The repeat distance between water molecules of 3.831Å is equivalent to the unit cell *a* axis length; C: Distances of hydrogen bonding interactions in the galangin crystal structure.

The galangin crystal grown after a few days from an ethanol solution showed color variation: it was clear when observed in one direction but reddish-yellow when observed at an approximate perpendicular direction. A data crystal was chosen (Fig 1) and an APEX2 DUO platform X-ray diffractometer from Bruker Advanced X-ray Solutions was used to obtain X-ray data measurements at 125 K. The crystal structure was solved and refined using ShelX programs [19]. Views of the crystal structure down the crystallographic axes, *a*, *b* and *c* are shown in S1 Fig. Torsion angles show the chromone moiety rings, A and C in Fig 2A, are in the same plane, while the phenyl ring, designated B, is rotated -45.1(2° degrees off that plane. An analysis of 852 flavone structures in the CCSD shows that this value is uncommon (the mean torsion angle value is -11°) [28].

Although galangin was crystallized from ethanol, a water molecule is present in the asymmetric unit. The water molecules function as hydrogen bonded bridges between layers of galangin molecules. Additionally, the distance between water oxygen atoms (viewed down the *a*-axis) is equal to the crystallographic *a*-axis (Fig 2B). The hydrogen bond parameters are listed in Table 2 and shown in Fig 2C. Besides these strong interactions, the crystal structure of galangin highlights the strong offset stacking between layers of galangin molecules (S2 Fig).

**Table 2:** Hydrogen bonds for galangin [Å and °].

D-H…A	d(D-H)	d(H…A)	d(D…A)	<(DHA)
O(3)-H(3)…O(2)#1	0.84(2)	2.02(2)	2.7352(15)	143(2)
O(3)-H(3)…O(2)	0.84(2)	2.28(2)	2.7391(15)	114.5(19)
O(4)-H(4)…O(2)	0.89(2)	1.85(2)	2.6405(15)	147.2(19)
O(5)-H7…O(1W)#2	0.88(2)	1.80(2)	2.6796(15)	176(2)
O(1W)-H(1W)…O(4)#3	0.87(2)	2.05(2)	2.8938(16)	163(2)
O(1W)-H(2W)…O(3)#4	0.86(2)	2.01(2)	2.8152(17)	155(2)

Symmetry transformations used to generate equivalent atoms:

^#1^ -x+1,-y+2,-z+1

^#2^ x-1,y,z

^#3^ -x,-y+1,-z+1

^#4^ x,y-1,z

The results from our experimental RRDE studies show that galangin has very strong antioxidant activity (described later) towards the superoxide radical anion and are compatible with earlier work using the DPPH method to evaluate galangin radical scavenging ability [29]. The galangin crystal structure with its rich network of hydrogen bonds and stacking interactions supports this conclusion. We then sought to explain this strong antioxidant behavior by using computational methods to explore galangin scavenging behavior with the superoxide radical anion.

### Antioxidant activity of galangin studied using DFT

**Fig 3:**
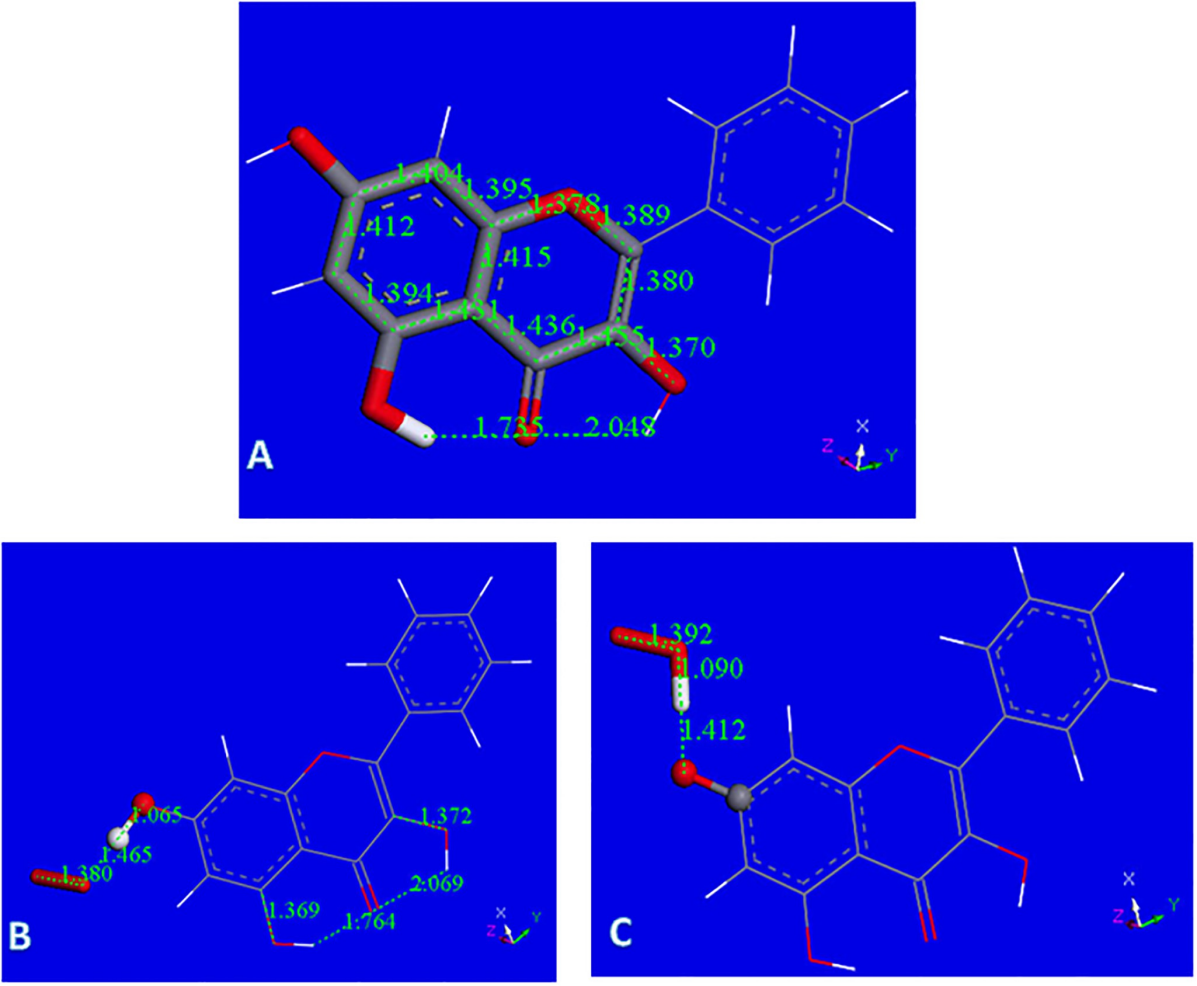
A: Galangin geometry obtained from X-ray coordinates after DFT minimization; B: Initial van der Waals separation between H7 and superoxide, 2.60 Å, decreases to 1.465 Å after DFT minimization, while hydroxyl separation is still a bond length, 1.065 Å. Superoxide: stick style; galangin 7-OH: ball and stick style; C: H7 was eliminated from the hydroxyl and the remaining species, the galangin radical, was DFT minimized and later placed 2.60 Å from HO_2_ for further geometry minimization. Fig 3C shows the converged energy minimum: the H atom of HO_2_ is not recaptured by the galangin radical, 1.412 Å. The energy of this minimum is 1.7 kcal/mol higher than that in Fig 3B, suggesting H7 of galangin is not captured by superoxide.

**Fig 4:**
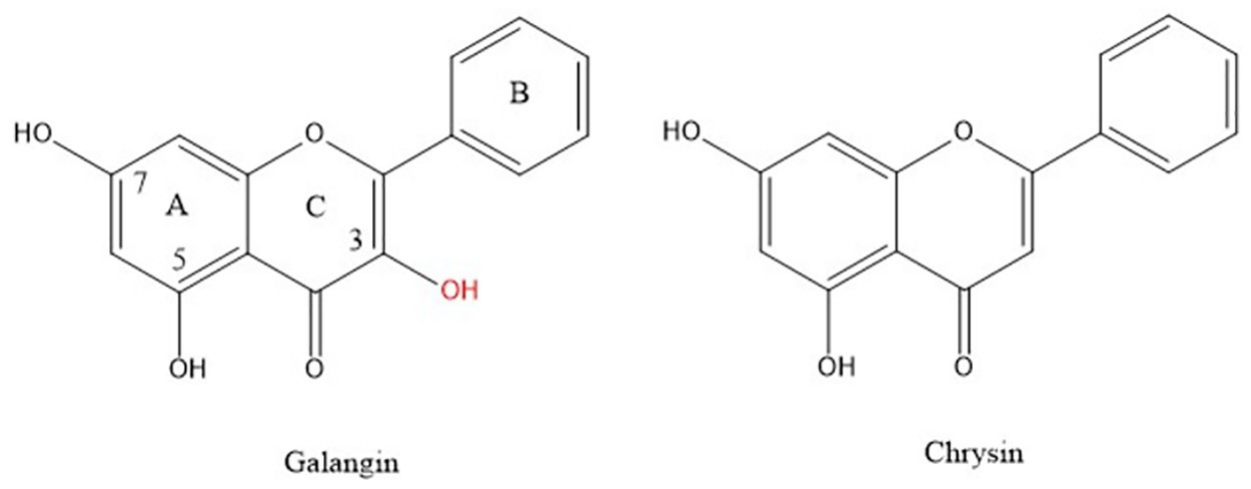
Galangin, 3,5,7-trihydroxyflavone, and chrysin, 5,7-dihydroxyflavone, are important components of propolis.

Galangin X-ray coordinates were input into Materials Studio program DMOL^3^, and Fig 3A shows relevant distances obtained after geometry optimization. This structure was obtained as an energy minimum, i.e. having only positive frequencies after vibrational analysis calculation; these distances will be of interest for comparison with the structural changes occurring after scavenging the superoxide radical. As will be described later, galangin is a good scavenger of superoxide and here we propose a potential mechanism of scavenging. The common mechanism by which polyphenols scavenge radicals is through H-atom transfer from a polyphenol hydroxyl to the radical. In galangin, there are 3 potential hydrogen atoms donors from the hydroxyl groups located in position 3, 5 and 7. Initially, one might imagine that the 7OH would be favored for this process, since the H atoms in the remaining hydroxyls of galangin form strong intramolecular H-bonds with the O(carbonyl), making these H atoms more difficult to extract from the polyphenol. Fig 3B shows the result of DFT geometry optimization for superoxide separated from H7, initially set as 2.60 Å, which is the distance corresponding to the sum of van der Waals distances for O and H atoms. It is seen that H7 is not captured by superoxide radical. However, this result may be due to an energy barrier and so we minimized the potential product to exclude that possibility: HO_2_ was placed at van der Waals separation from the H7 excluded radical galangin, and the whole system becomes a radical species. This results in no recapture of H7 by the galangin radical, Fig 3C. Comparing energies of the resulting product structures shown in Fig 3C and 3B, we see that the latter is 1.7 kcal/mol lower and the positive ΔG indicates no capture of H7 by galangin. S3 Fig shows the result of geometry minimization for an initially separated proton (2.60 Å) from the O_2_ moiety of Fig 3B, that results in no formation of H_2_O_2_. This is in contrast with the behavior of chrysin [22], where H7 was transferred to superoxide and evolved towards H_2_O_2_ formation. Fig 4 shows the 2D structures of galangin and chrysin. It is interesting that the presence of an additional 3-hydroxyl in galangin can preclude this type of scavenging.

**Fig 5:**
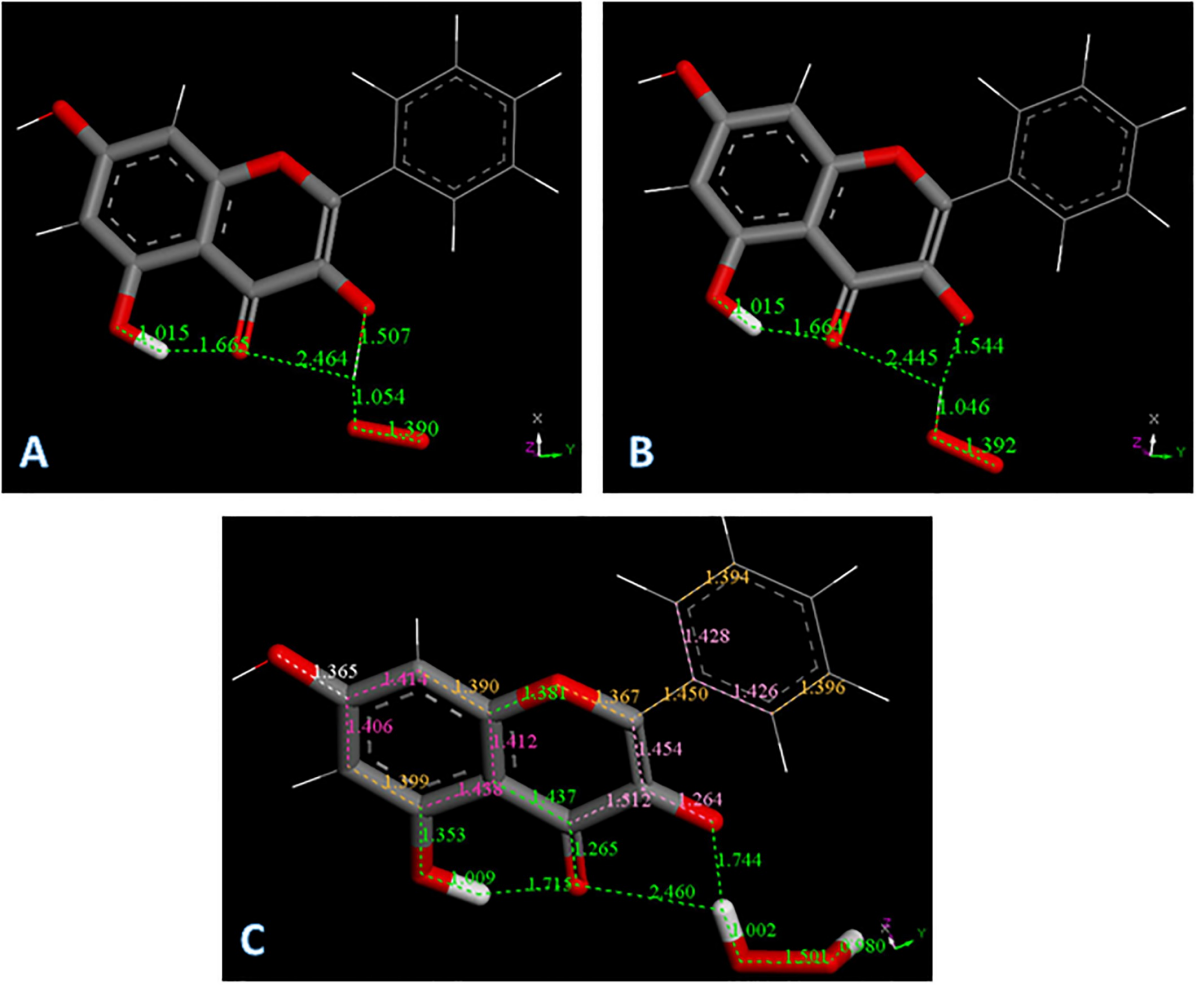
A: Converged DFT minimum for superoxide attack on galangin H3. The galangin O-H3 bond distance elongates to 1.507 Å and H3 advances to establish a bond distance to superoxide, 1.054 Å; B: Converged DFT minimum for initial van der Waals contact between HO_2_ and the radical obtained after excluding H3 from galangin. The galangin radical does not recapture H3, 1.544 Å; C: The galangin-superoxide complex shown in Fig 5A is van der Waals placed nearby a proton. This is a neutral radical that after minimization shows formation of H_2_O_2_ plus the galangin derivative radical, which locates the unpaired electron in the polyphenol.

However, it is known that the presence of a 3-hydroxyl in the flavonoid system induces antioxidant activity [30]. Here we show that the interaction between superoxide and this hydroxyl substituent in galangin confirms this experimental fact. Minimization of the van der Waals approached superoxide to H3(hydroxyl), Fig 5A, converges to a structure very similar to the one approaching HO_2_ and the H3 excluded galangin radical, Fig 5B. More importantly, the energies of these 2 minima are exactly the same (ΔG = 0.01 kcal/mol). We conclude that the initial interaction between galangin H3 and superoxide generates a complex. It is interesting to analyze the outcome when a proton approaches this complex. From the structural complex created in the related minimization, shown in Fig 5C, it is seen that H_2_O_2_ is formed.

The galangin-superoxide complex shown in Fig 5A was van der Waals placed nearby a proton. This is a neutral radical that after minimization shows formation of H_2_O_2_ plus the galangin derivative radical, which locates the unpaired electron in the polyphenol, Fig 5C. The C3-O3 bond, 1.264 Å, becomes shorter than in galangin, (1.370 Å, Fig 3A), and very similar to the O = C4 bond, 1.265 Å. This results in rearrangement of nearby bonds: C2-C3 = 1.454 Å and C3-C4 = 1.512 Å, which are longer than in galangin 1.380 Å, and 1.455 Å, respectively, Fig 3A. C2-C1’ = 1.450 Å and O1-C2 = 1.367 Å, are shorter when compared with galangin 1.473 Å and 1.389 Å, respectively in Fig 3A. Also, the aromatic B ring now shows loss of aromatization as C2’-C3’ (1.394 Å) and C5’-C6’ (1.396 Å), are shorter than the other C-C bonds in the ring, C1’-C2’ (1.428 Å), C5’-C1’ (1.426 Å). We can consider this radical having an extended conjugation. Ring A shows 2 bonds (yellow 1.390 Å and 1.399Å) shorter than lengthened bonds, (purple 1.414 Å, 1.406 Å, 1.438 Å and 1.412 Å), but this effect is already present in free galangin, Fig 3A and in the X-ray crystal structure (1.381(2) Å and 1.377(2) Å).

**Fig 6:**
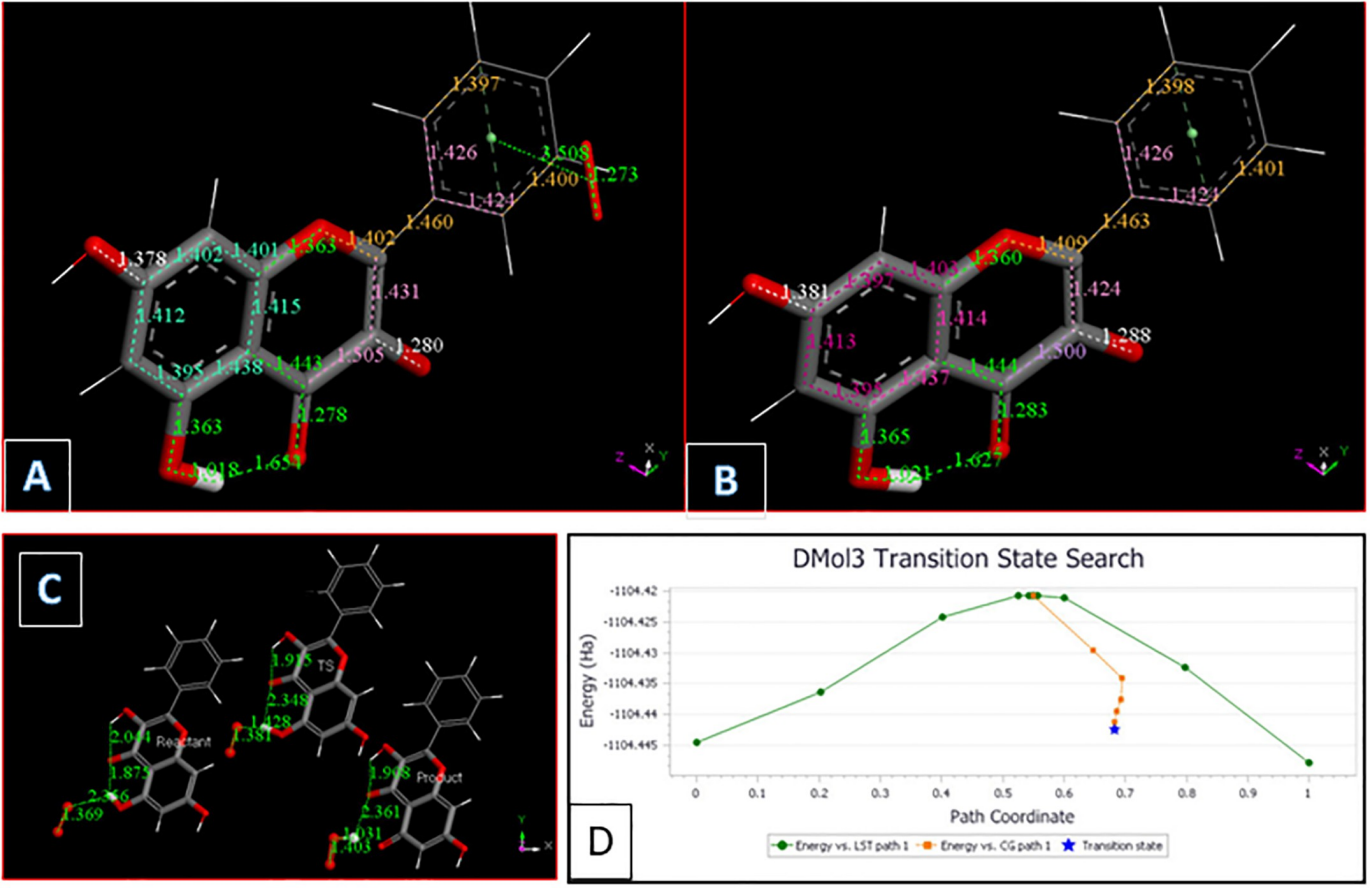
A: The π-π approach of a superoxide to galangin ring B allows for ring capture of its unpaired electron. O-O bond distance becomes similar to that in molecular oxygen, 1.273 Å, in contrast with the approached superoxide O-O, 1.373 Å. B: From previous Fig 6A O_2_ is eliminated and DFT minimization is performed; C: Reagent, TS and Product for galangin H5 extraction by superoxide. ΔG = -2.067 kcal/mol, E(barrier) = 1.3 kcal/mol; D: TS profile for galangin H5 extraction by superoxide. ΔG = -2.067 kcal/mol, E(barrier) = 1.3 kcal/mol.

Using our computational results, we provide mechanistic evidence that galangin can act as a superoxide dismutase (SOD) mimic. From Fig 5C, H_2_O_2_ was excluded, and superoxide π-approached to ring B, which was chosen because it has some quinone characteristics and so likely to be a good target for capturing the superoxide unpaired electron. Indeed, the superoxide electron is captured by the ring, while O_2_ is formed (O-O bond distance = 1.273 Å) but the latter does not penetrate galangin π system, as its separation from ring B is still similar to van der Waals value, 3.508 Å, Fig 6A. Hence, molecular oxygen was eliminated, and the residual structure minimized, Fig 6B. Finally, a proton was van der Waals approached to galangin O3 and, upon minimization, galangin was reformed. This suggests galangin acts as a SOD mimic for scavenging superoxide with H3 supporting reaction (a), corresponding to SOD activity:

$${2\;\text{O}}_{2}{•}^{-}+2{\text{H}}^{+}➜{\text{H}}_{2}{\text{O}}_{2}+{\text{O}}_{2}$$ (a)

It is seen that 1) H_2_O_2_ is easily detached from Fig 5C, as O_2_ is from Fig 6A, and these molecules are the products of reaction (a). In addition, after eliminating O_2_, the arrival of a reactant proton from reaction (a), regenerates galangin, which is ready for another catalytic cycle. The second reactant proton from reaction (a) is the one added to HO_2_ in Fig 5B and used to form H_2_O_2_, Fig 5C. This cycle is summarized in Scheme 1.

**Scheme 1**. **Galangin is regenerated after reacting with 2 protons and 2 superoxide radicals**. (1) Incoming superoxide (red) interacts with H3; (2) H3 is sequestered, forming HO_2_; (3) a proton arrives and adds to HO_2_ forming, and eliminating, H_2_O_2_ (light blue); (4) A second incoming superoxide (green) interacts π-π with galangin ring B; (5) the superoxide donates its electron and O_2_ is released (light blue); (6) an incoming proton interacts with O3, re-establishing the galangin 3OH hydroxyl group and thus reforming galangin.

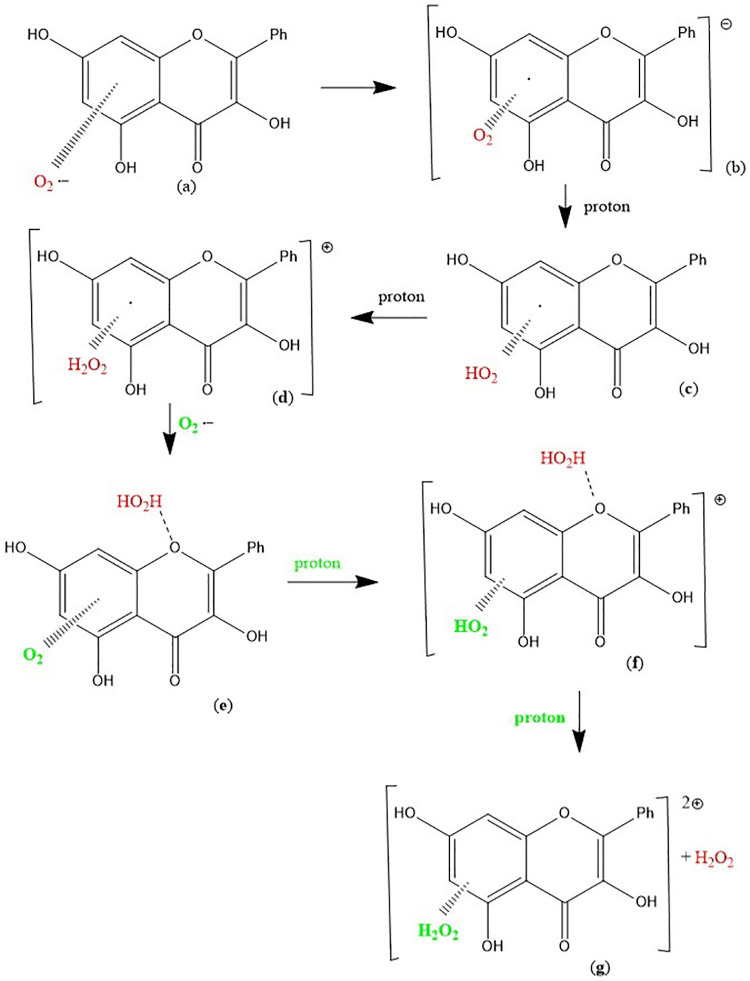


Finally, we explore if hydroxyl H5 can also detach from galangin when approached by superoxide, by performing DFT-D minimizations. Although there is some shortening between H5 and superoxide, it is much weaker than that seen for H3, and additional approach by a proton does not induce H_2_O_2_ formation (not shown). Hence, we studied the behavior of the potential product, which is an HO_2_ moiety interacting with galangin after H5 exclusion. The energy of this product is lower and a TS was explored. Fig 6C and 6D, show that the capture of H5 by superoxide is feasible: ΔG = -2.067 kcal/mol, E(barrier) = 1.3 kcal/mol.

We conclude that, abstraction of a galangin H atom by superoxide is feasible for hydroxyl hydrogens H3 and H5, with H3 slightly favored because it is a thermodynamically spontaneous process, whereas H5 needs to overcome an energy barrier. However, this H5 barrier is easily affordable (1.3 kcal/mol). Also, in contrast with chrysin [22], H7 galangin is not captured by superoxide.

The antioxidant ability of flavonoids has been studied fro some time [11–17]. Recently, computational studies on flavonoid derivatives, including galangin and quercetin, have focused primarily on the importance of hydroxyl groups in exocyclic phenyl ring B (which galangin does not have) and pyrone ring C (the C-3 hydroxyl group) present in both galangin and quercetin [18]. Our DFT results are in agreement with findings in this study, than the hydroxyl H3 atom is slightly favored for hydrogen abstraction and antioxidant activity, over hydroxyl H5.

**Fig 7:**
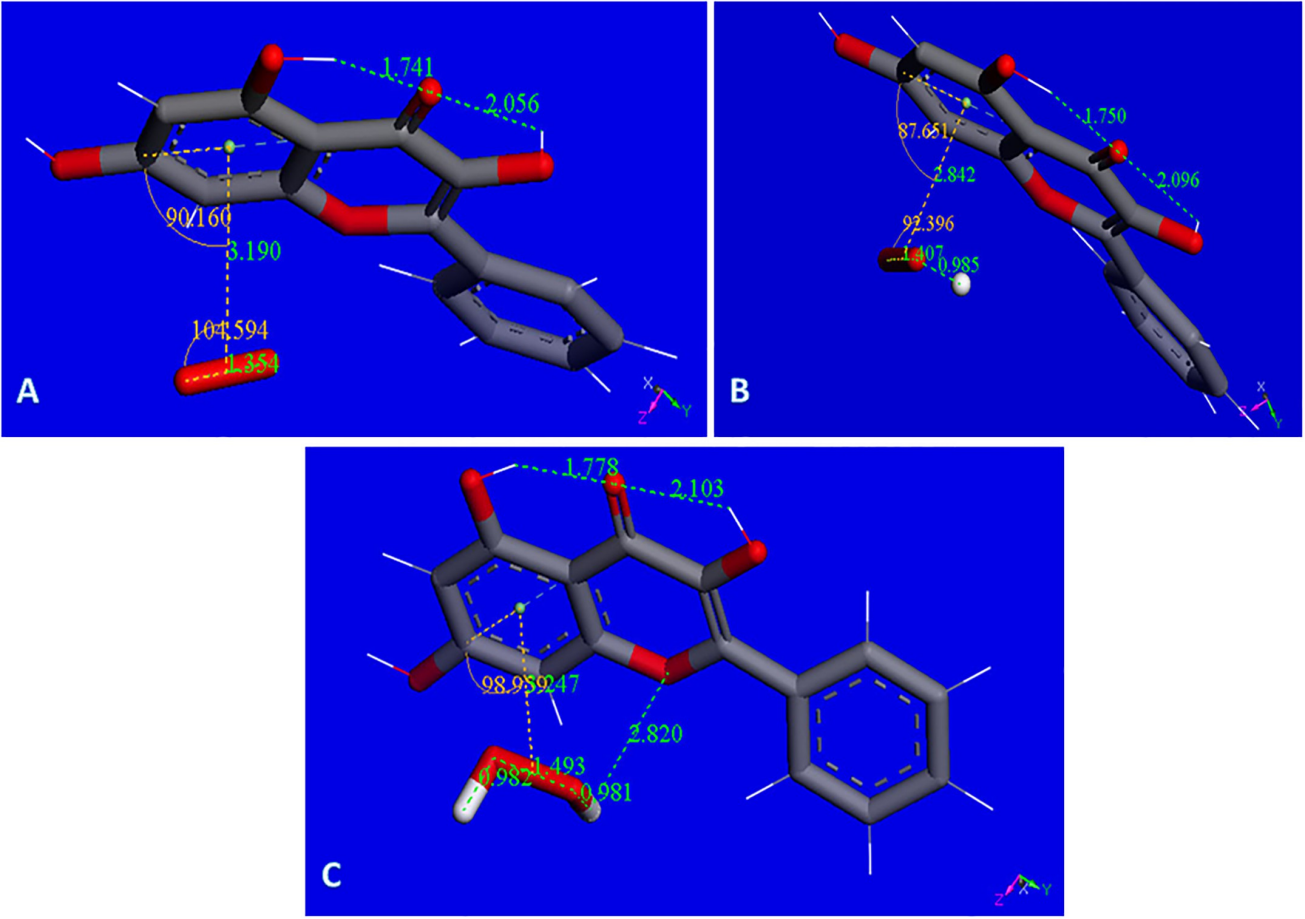
A: Galangin accepts a π-π approach by superoxide. Initially van der Waals separated molecules, 3.50 Å, are geometrically optimized. Both reagents become closer with centroid-centroid separation stabilized 3.190 Å apart. There is a slight shortening of O-O bond distance in superoxide, 1.354 Å, from the original 1.373 Å; B: Galangin π–π superoxide from previous Fig 7A is now approached by a proton that, after minimization, becomes stabilized by formation of an O-H bond, 0.985 Å, while superoxide O-O separation is lengthened, 1.407 Å (it was 1.354 Å in Fig 7A); C: From previous Fig 7B, an additional proton is placed 2.60 Å from HO_2_. Upon minimization H_2_O_2_ is formed, however, it is in a complex with the galangin ring, as shown by the centroid-centroid separation of 3.247 Å, shorter that the 3.50 Å π–π van der Waals separation.

**Fig 8:**
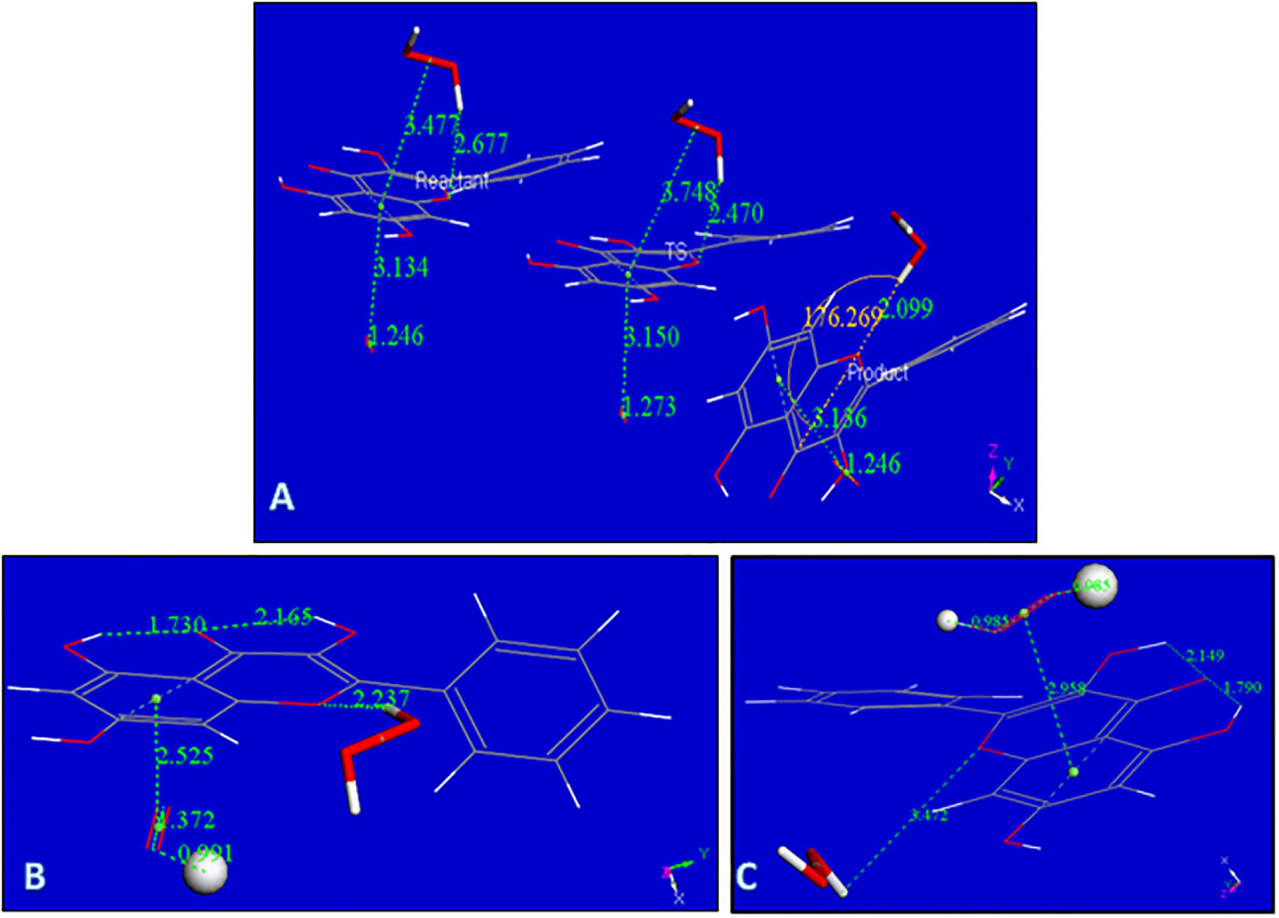
A: Rearrangement of H_2_O_2_ moiety as described by the Transition State search (TS): left, reagent; center TS; right, product, are displayed; B: From the product of Fig 8A proton is captured by the O_2_ moiety, total charge is 1; C: From Fig 8B an additional proton, small ball size, is captured by the HO_2_ moiety forming H_2_O_2_, while the other H_2_O_2_ located in the polyphenol plane, detaches from galangin, 3.472 Å. Total charge of this radical species is 2.

Our previous investigations on superoxide scavenging by embelin [31] emodin [32], and chalcones [33] described an additional mode of sequestering superoxide, through the π-π interaction between superoxide radical and the polyphenol. This scavenging mechanism is suggested for galangin as well, since, as described later in RRDE results, galangin is experimentally found to be an excellent scavenger of superoxide, and additional options need to be considered. Computational results seen in Fig 7A show the related minimization structure where centroid-centroid separation stabilized at 3.190 Å, from an initial van der Waals separation of 3.5 Å. The complex incorporates a proton captured by the O_2_ moiety, Fig 7B, and an additional proton generates the H_2_O_2_ moiety, still coordinated to the polyphenol, Fig 7C. An additional calculation for interaction to a 2^nd^ superoxide radical, entering from the opposite side of H_2_O_2_, (S4 Fig, the input configuration) shows the incoming group stabilized with a centroid-centroid separation of 3.134 Å. The original separation between superoxide O atoms becomes much shorter, 1.246 Å, corresponding to the expected length of a O_2_ molecule, but still bound to the polyphenol. It is clear that the superoxide unpaired electron is now located within the polyphenol structure and this species is *not* a radical, as the number of electrons is now *even* due to 2 superoxides incorporated into the complex. It is noteworthy that one H from H_2_O_2_ now is within H-bonding distance (2.820 Å) to the polyphenol O1. To analyze the corresponding potential H_2_O_2_ transfer to the polyphenol plane, a transition state calculation was performed after observing that the product has lower energy than the reagent, Fig 8A and 8B.

Fig 8A includes the minimum geometry reached after minimization of S4 Fig. The H_2_O_2_ moiety protrudes towards ring C, with a H-bond distance between O1 (galangin) and H(H_2_O_2_) of 2.677 Å, suggesting a potential rearrangement. This is confirmed with a transition state search where the product is shown on the right in Fig 8A, and the corresponding TS is in center of Fig 8A. ΔG is -0.04 kcal/mol and E(barrier) of 3.9 kcal/mol. S5 Fig shows the TS energy profile.

**Fig 9:**
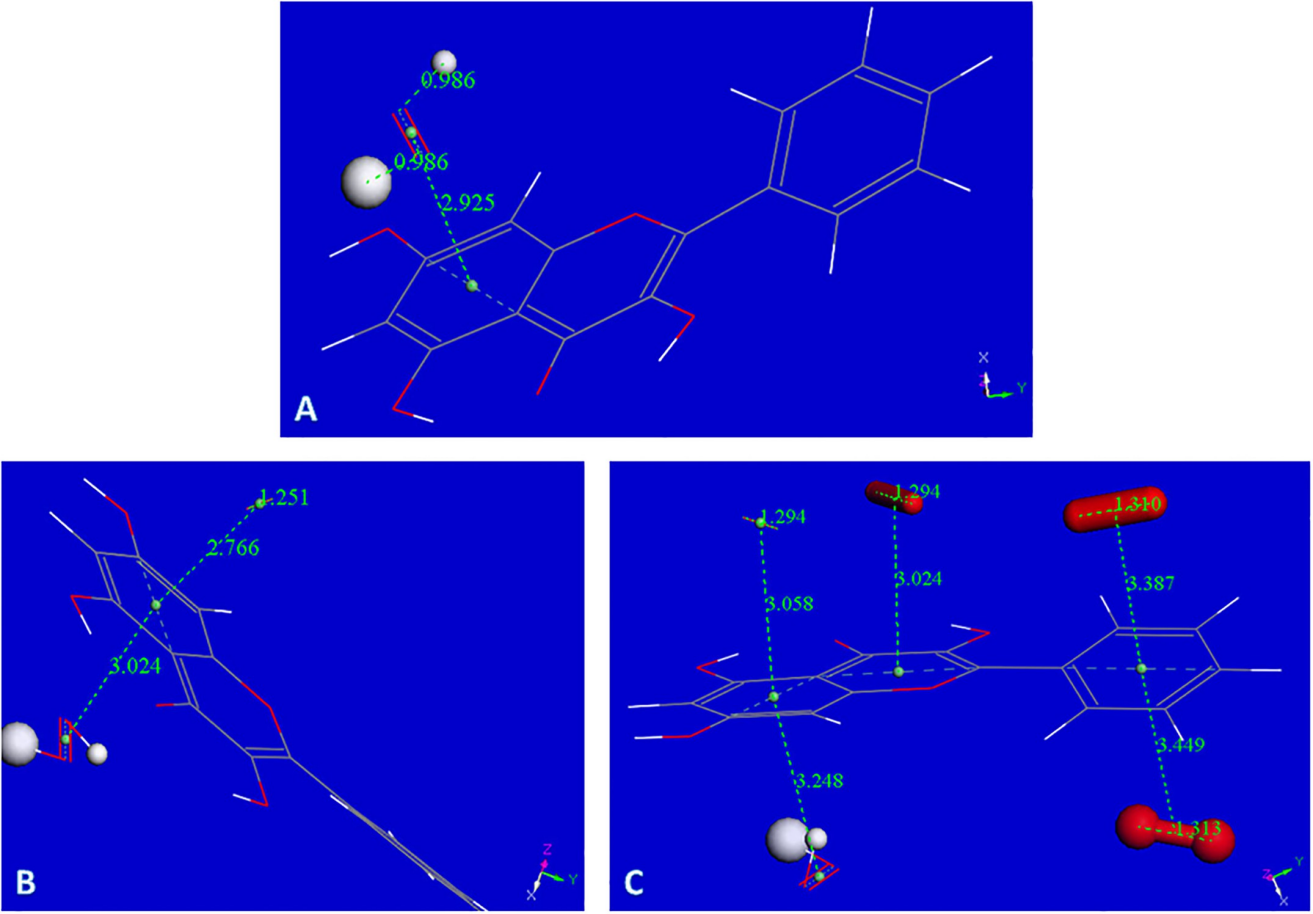
A: Minimum reached after excluding H_2_O_2_ from Fig 8C. Total charge is still 2; B: Superoxide entering trans to H_2_O_2_ moiety, total charge 1. This is a non-radical species and the O-O separation for the newly arrived superoxide, 1.251 Å, is much shorter than the original 1.373 Å, and similar to O-O bond length in O_2_; C: After the arrangement shown in Fig 9B, three additional superoxide radicals are incorporated above and below the aromatic plane of galangin.

Fig 8B shows the arrival of a proton to the O_2_ moiety of the product just described. After incorporation of another proton into the HO_2_ unit, a 2^nd^ molecule of H_2_O_2_ is formed while the first H_2_O_2_, located in the polyphenol plane detaches, with O(1)---H(H_2_O_2_) separation of 3.472 Å, Fig 8C. After elimination of the first H_2_O_2_ molecule, it is possible to incorporate an additional superoxide, Fig 9A and 9B.

Attempts to explore if the newly formed O_2_ species, trans to H_2_O_2_, or H_2_O_2_ itself, are able to detach from the Fig 9B complex failed, although additional superoxide radicals were incorporated as seen in the arrangement of Fig 9C. Indeed, the complex shown in Fig 9C contains the maximum number of superoxide radicals captured by galangin in this study. It is strongly suspected that if additional protons are available they may be incorporated to stabilize further H_2_O_2_ units. This DFT study is summarized in the following Scheme 2.

**Scheme 2**. The π-π scavenging of superoxide by galangin: (a) Superoxide interacts with galangin ring A (π-π approach); (b) the unpaired electron enters the galangin ring; (c) (d) two protons are incorporated within the O_2_ moiety forming H_2_O_2_; (e) a 2^nd^ superoxide is incorporated, (f) a proton captured by O_2_ forms a HO_2_ moiety; (g) after an additional proton the original H_2_O_2_ (red) detaches from O-1 galangin position.

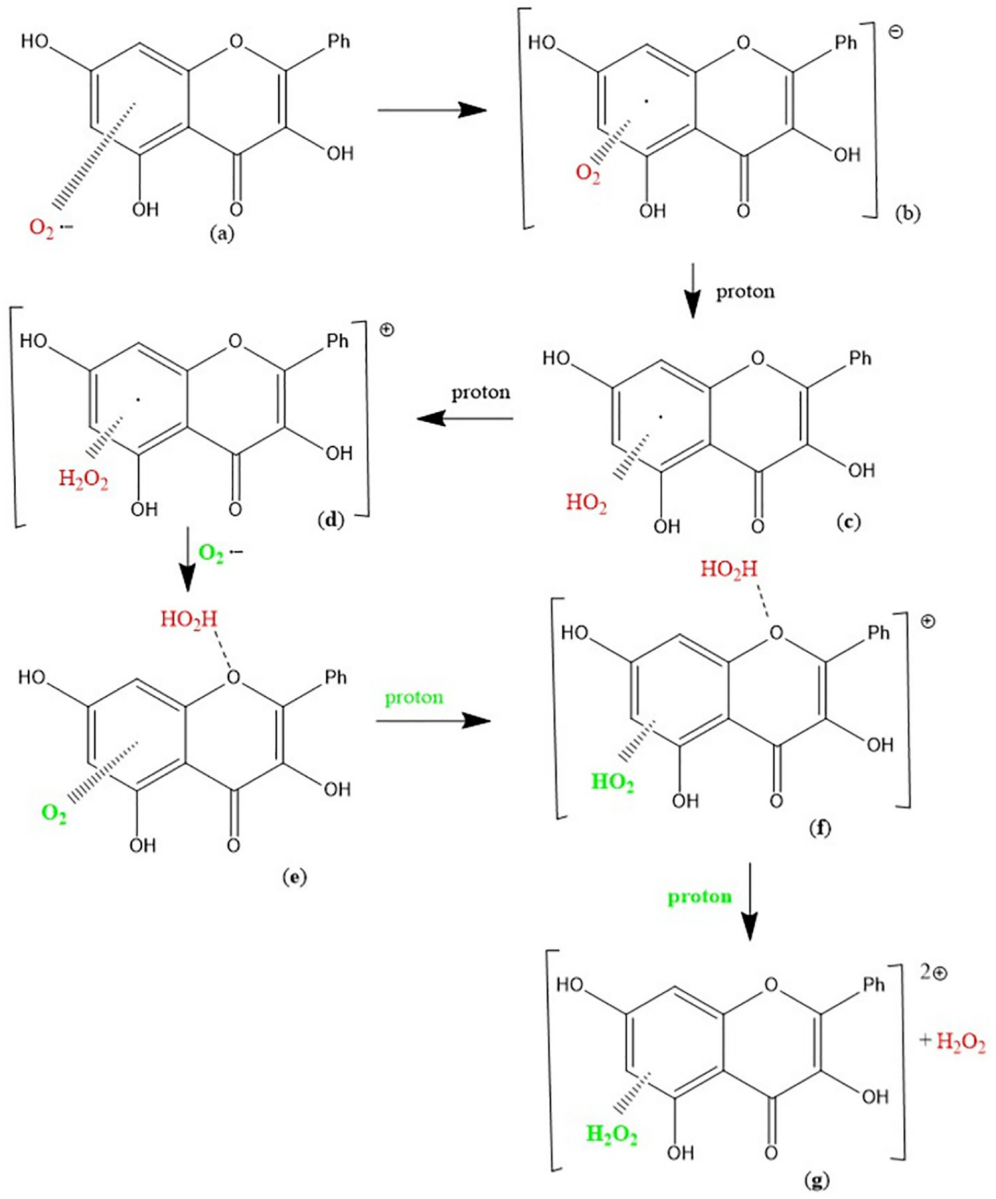


We see that the π-π approach of superoxide to galangin is favored and even 4 superoxide radicals can be stabilized along with a H_2_O_2_ molecule on galangin (Fig 9C), with total charge = -2, which is surprisingly high. This complex is a result of 5 incorporated superoxide and 2 protons, but before formation of this complex, H_2_O_2_ formation and release was described. Thus, the number of superoxide anions associated with galangin in this π-π superoxide-galangin study is six, which when added to 4 protons, defines the final charge of the complex (-2) shown in Fig 9C.

From this DFT study, we conclude that 1) galangin hydroxyls H3 and H5 can be captured by superoxide, suggesting galangin acts as a mimic of SOD catalysis; 2) H7 is not captured by superoxide; 3) galangin π-π interactions can incorporate several superoxide radicals, and form a detached H_2_O_2_ product. These conclusions are further discussed when analyzing the RRDE experimental results of galangin scavenging of superoxide, *vide infra*.

### RRDE

**Fig 10:**
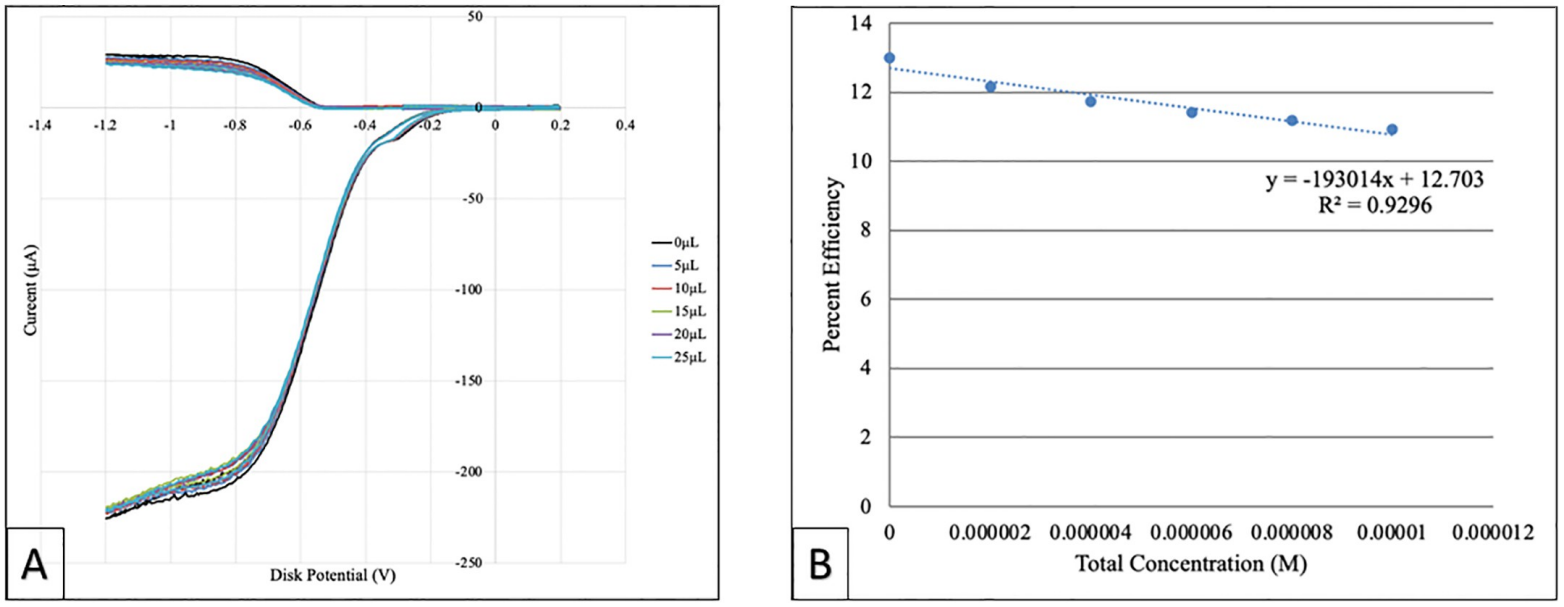
A: A total of 5 runs of galangin were performed in this study. This CV compilation shows the initial blank, black color, and colored aliquots added of galangin; B: Antioxidant Efficiency of galangin, as function of molarity.

The antioxidant activity of galangin was determined based on its superoxide radical scavenging ability that was measured using a protocol developed in our lab [22]. In this cyclic voltammetry method superoxide is developed *in situ* by bubbling O_2_ into the electrovoltaic cell containing anhydrous DMSO solvent, see details in Experimental section. After the blank experiment, aliquots taken from a stock 0.02M galangin solution, were added to the voltaic cell. Fig 10A shows voltammograms of blank and all runs for both electrodes. The lower part of these voltammograms develops at the disk electrode, where O_2_ incorporates an electron to generate superoxide O_2_•^-^. The opposite effect, oxidation of O_2_•^-^, takes place at the ring electrode, which has a fixed potential enough positive for this action, upper part. As scavenger aliquots are added, they consume part of the superoxide and the ring disc detects less superoxide, lowering the current. The collection efficiency is shown in Fig 10B, for all data, showing the decrease of superoxide. All scavengers studied by us so far behaved in a linear manner for the initial aliquots used. Beyond the initial aliquots, 2 possibilities were seen: (1) complete linear behavior, as in, chrysin [22], eriodictyol [22], BHT [31], emodin [32], celastrol [34] or (2) initial linear behavior followed by asymptotic performance, such as in quercetin [22], embelin [31] and clovamide [35]. In the present study, we also calculated a linear trend for all data, with regression factor of 0.9296, whereas the initial 3 data (using the blank plus 2 initial added aliquots) have a linear regression factor of 0.9635, slightly better, but still not ideal. Since we find that several superoxide anions are incorporated in one galangin unit, Fig 9C, galangin is a very efficient scavenger, and indeed this is demonstrated by the slope in Fig 10B, -19x10^4^. In fact, when comparing with previous studies following the same protocol, quercetin [31], (slope = -6.0x10^4^) was the radical scavenger showing the steepest slope; in this study, galangin shows an even stronger superoxide scavenging capacity than quercetin. This is in contrast with calculations reported by Spiegel et al. [18] where thermodynamic parameters showed quercetin as a better radical scavenger, and which was explained as due to the catechol moiety present in phenyl ring B of quercetin.

Our DFT study demonstrates the strong capability of galangin to capture superoxide radicals via π-π interaction. This is in agreement with our experimental RRDE showing galangin to be a better antioxidant than quercetin by having a steeper slope (Fig 10B) compared with quercetin. It is interesting that the commercially used antioxidant, BHT, has a much milder slope, -1.6x10^3^, [31].

**Fig 11:**
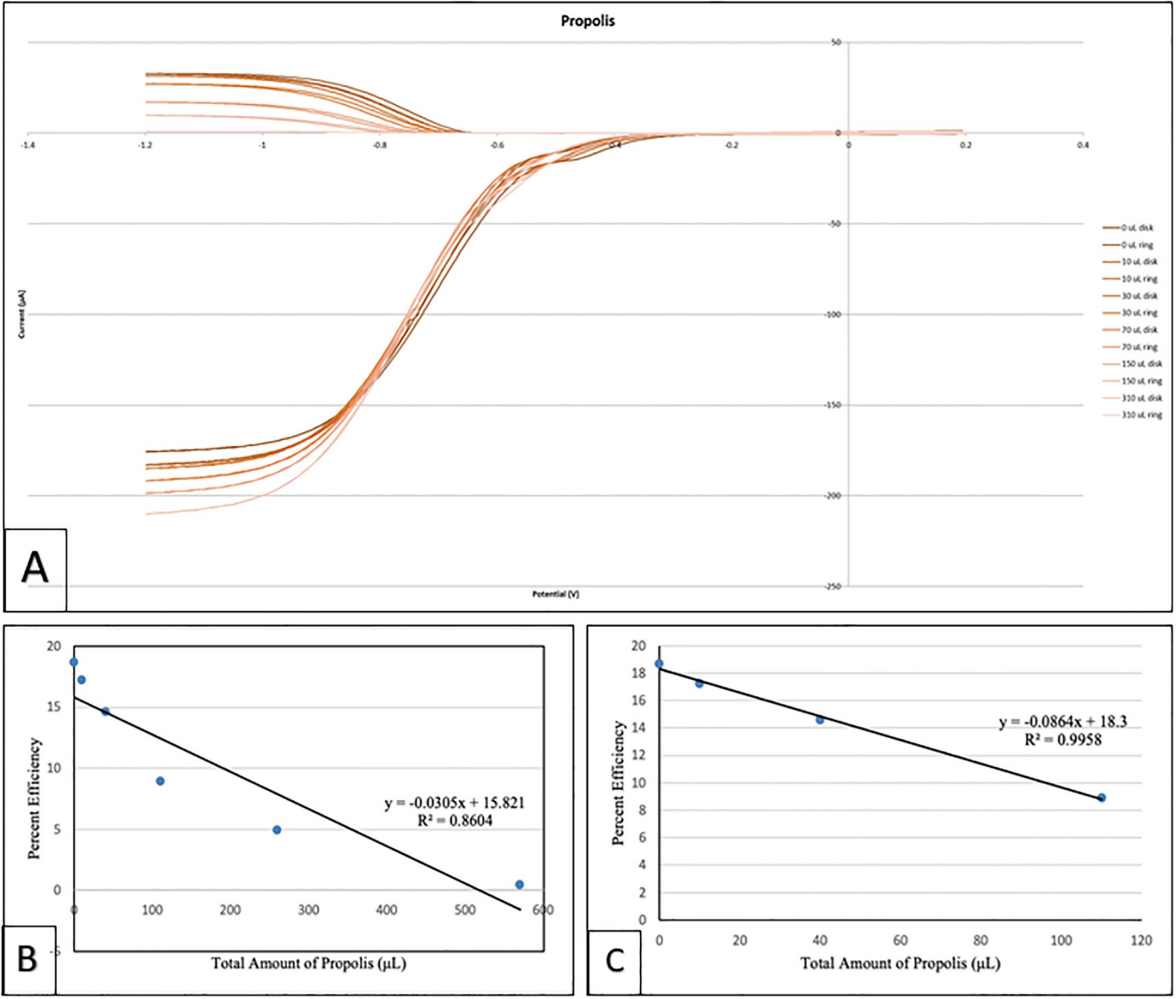
A: Cyclovoltammograms of propolis when scavenging the superoxide radical, B: Collection efficiency of propolis; linear behavior considering all runs. The last run shows almost complete elimination of superoxide; C: Linear behavior of propolis considering the first 4 data on Fig 11A.

Propolis was also analyzed using the RRDE method. Fig 11A shows the related voltammograms and Fig 11B shows the antioxidant efficiency of propolis when scavenging the superoxide radical where the x-axis denotes the total volume of added aliquots; the linear form shows a non-ideal regression factor, 0.8604. Fig 11C only considers the initial 4 points of Fig 11B shows a linear behavior with an excellent regression factor, 0.9958. When comparing with our previously described RRDE analysis of olive oil [36], which is also a mixed substrate, propolis shows higher antioxidant activity, as shown by an almost complete elimination of superoxide after the last aliquot.

## Conclusions

Results from cyclic voltammetry using a ring disk electrode (RRDE), show that galangin is a powerful scavenger of the superoxide radical anion. This is compatible with the galangin crystal structure that shows a rich network of hydrogen bonds and stacking interactions. Using DFT methods, π-π interaction between superoxide and galangin are further confirmed; these interactions are in part responsible for superoxide oxidation and capture of the superoxide electron by the aromatic rings of galangin. Additionally, abstraction of galangin H3 and H5 by superoxide is also demonstrated and associated with SOD action by galangin. This SOD action may be responsible for the RRDE steeper slope of galangin compared with quercetin [22]. When analyzing propolis, RRDE results show very strong antioxidant capability, including almost complete elimination of superoxide from the electrochemical cell. Since propolis contains other scavengers besides galangin, a main component of propolis, the total amount of effective compounds in the propolis contribute to the antioxidant activity.

## Supporting information

S1 Fig Unit cell packing as viewed down *a*-axis, down *b*-axis and down *c*-axis, from left to right. (JPG)

S2 Fig Galangin unit cell offset stacking at 3.356 Å. The diagram on the right is approximately perpendicular to the view on left.
(JPG)

S3 Fig From previous Fig 4C a proton was placed at van der Waals separation from the superoxide (2.60 Å). After geometry optimization this proton went further away, 2.890 Å. Thus, this proton does not induce H_2_O_2_ formation, a potential product resulting from scavenging of superoxide by polyphenols.
(TIF)

S4 Fig In this DFT calculation, an additional superoxide is placed on the opposite side of the galangin ring A from the H_2_O_2_, at initial separation of 3.50 Å. (TIF)

S5 Fig TS profile of reaction in Fig 8A. (TIF)
