# Correction: Antioxidant properties of bee propolis and an important component, galangin, described by X-ray crystal structure, DFT-D and hydrodynamic voltammetry

**DOI:** 10.1371/journal.pone.0314810

**Published:** 2024-11-27

**Authors:** Francesco Caruso, Molly Berinato, Melissa Hernandez, Stuart Belli, Christopher Smart, Miriam Rossi

Scheme 1 is a duplicate of Scheme 2. Please view the correct Scheme 1 here.

**Scheme 1:**
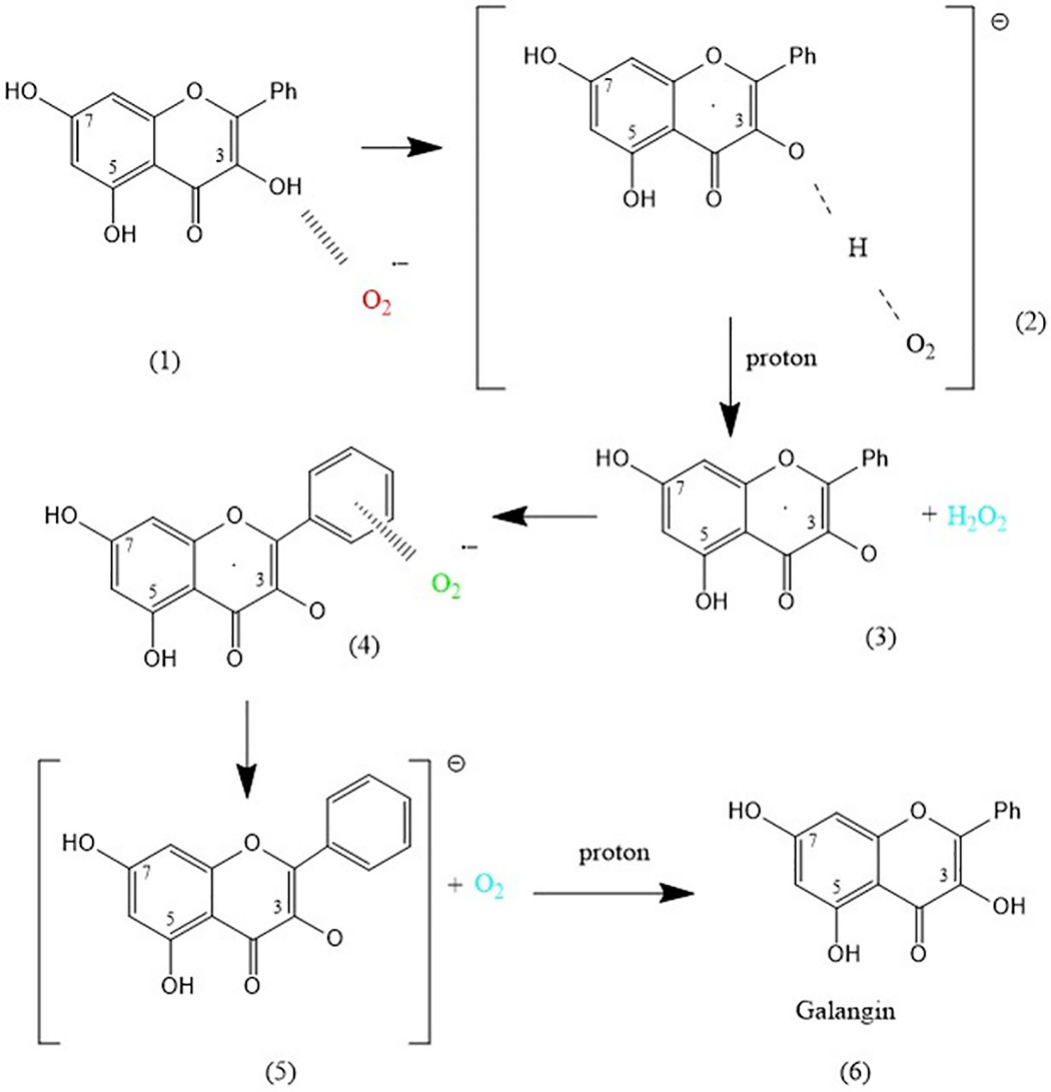


## References

[1] Caruso F, Berinato M, Hernandez M, Belli S, Smart C, Rossi M (2022) Antioxidant properties of bee propolis and an important component, galangin, described by X-ray crystal structure, DFT-D and hydrodynamic voltammetry. PLOS ONE 17(5): e0267624. 10.1371/journal.pone.0267624 35584109 PMC9116673

