# Supplementary figures and images for: Antioxidant properties of bee propolis and an important component, galangin, described by X-ray crystal structure, DFT-D and hydrodynamic voltammetry
Source: PLoS One. 2022 May 18;17(5):e0267624. doi: 10.1371/journal.pone.0267624 (PMC9116673; doi:10.1371/journal.pone.0267624)

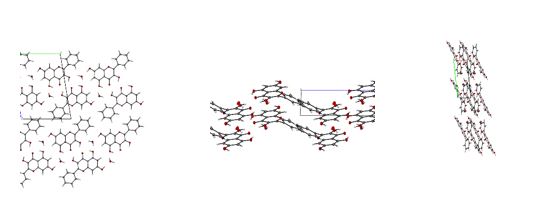

Supplement: S1 Fig — (JPG) [file pone.0267624.s001.jpg]

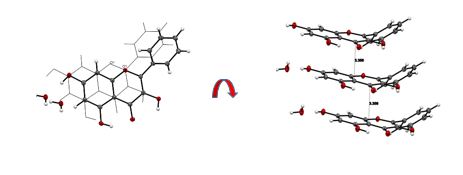

Supplement: S2 Fig — The diagram on the right is approximately perpendicular to the view on left. (JPG) [file pone.0267624.s002.jpg]

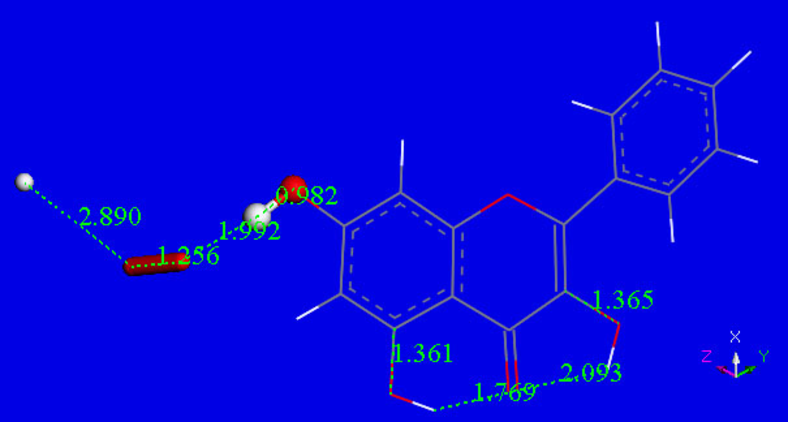

Supplement: S3 Fig — After geometry optimization this proton went further away, 2.890 Å. Thus, this proton does not induce H2O2 formation, a potential product resulting from scavenging of superoxide by polyphenols. (TIF) [file pone.0267624.s003.tif]

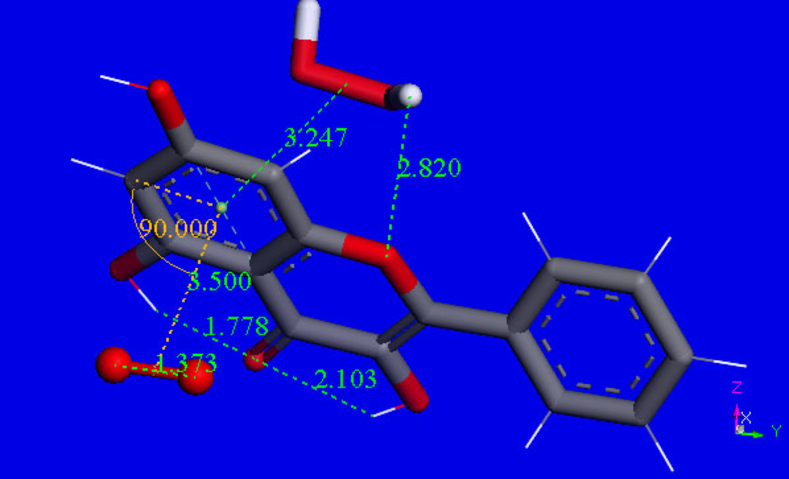

Supplement: S4 Fig — (TIF) [file pone.0267624.s004.tif]

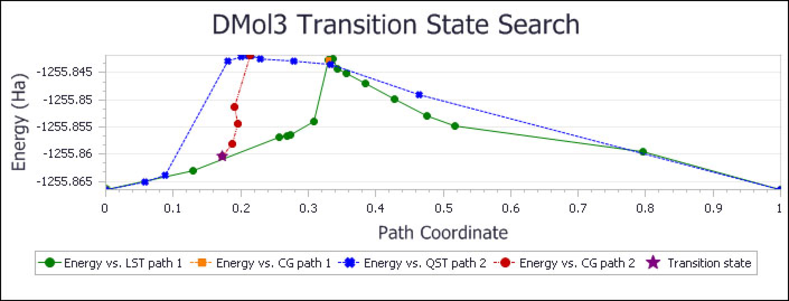

Supplement: S5 Fig — (TIF) [file pone.0267624.s005.tif]
